# Genomics in the era of COVID-19: ethical implications for clinical practice and public health

**DOI:** 10.1186/s13073-020-00792-9

**Published:** 2020-11-09

**Authors:** Gail Geller, Priya Duggal, Chloe L. Thio, Debra Mathews, Jeffrey P. Kahn, Lisa L. Maragakis, Brian T. Garibaldi

**Affiliations:** 1 Berman Institute of Bioethics, Johns Hopkins University, Deering Hall, Room 202, 1809 Ashland Ave., Baltimore, MD 21205 USA; 2 Johns Hopkins University School of Medicine, Baltimore, MD USA; 3 Johns Hopkins Bloomberg School of Public Health, 615 N. Wolfe St., Baltimore, MD 21205 USA; 4 Johns Hopkins School of Medicine Hepatitis Center, 855 N. Wolfe St. Rangos Room 533, Baltimore, MD 21205 USA; 5 Johns Hopkins School of Medicine Infection Control, 600 N. Wolfe St., Osler 425, Baltimore, MD 21205 USA; 6 Johns Hopkins University School of Medicine Pulmonology, 5501 Hopkins Bayview Circle, Baltimore, MD 21224 USA

**Keywords:** COVID-19, Ethics, Host genomics

## Abstract

Genomic studies of patients with COVID-19, or exposed to it, are underway to delineate host factors associated with variability in susceptibility, infectivity, and disease severity. Here, we highlight the ethical implications—both potential benefits and harms—of genomics for clinical practice and public health in the era of COVID-19.

## COVID-19, genomics, and ethics

The novel disease caused by the coronavirus SARS-CoV-2 (COVID-19) has resulted in over 46 million confirmed cases and over 1.2 million deaths worldwide, with the USA having the largest number of cases and deaths. COVID-19 is transmitted easily and quickly and is associated with a wide variability in severity of symptoms.

Evidence from other infectious diseases suggests that variability in host genomics influences susceptibility to infections, vaccine and treatment response, morbidity, and mortality [1]. Thus, human genetic variation will likely contribute to some of these outcomes with COVID-19. Genomic studies of patients diagnosed with and exposed to COVID-19 are underway to identify genetic similarities among those most at risk for severe outcomes and to guide development of therapeutics [2, 3]. However, prior to using such genomic data in clinical and public health decision-making, it is important to consider a number of ethical, legal, and social implications (ELSI). A recent paper highlights the societal trade-offs necessitated by COVID-19 between protecting health and risking discrimination and exclusion from public spaces [4]. Outside the context of COVID-19, we have recently argued for the importance of anticipating ELSI issues when conducting *research* on the genetics of infectious disease more generally [5, 6]. In this paper, we highlight the ethical implications of genomics for *clinical practice* and *public health* in the era of COVID-19 and derive a set of key questions that would need to be considered in this context (Table 1). Whereas our previous work considered “in principle” arguments in the context of hypothetical scenarios, COVID-19 provides an opportunity to move from theory to action in a very real context.

**Table 1:** Ethical questions in the application of genomics to COVID-19 management decisions

Clinical decisions Is it ethically acceptable …	to withhold scarce resources from patients with higher (or lower) genetic risk of mortality?
	to deny hospital admission to patients with higher (or lower) genetic risk of mortality?
	to use genetic information to make decisions about which patients are admitted to the ICU or put on a ventilator?
	to prioritize access to experimental treatments (which are in short supply) to those who are genetically at higher risk of serious disease?
	for visitation policies to be informed by genetic testing of family members to determine their risk of contracting or transmitting COVID-19?
	to mandate that adherence to DNR orders should be stricter for patients with higher genetic risk of mortality?
Workforce decisions Is it ethically acceptable …	for hospitals/ICUs to mandate genetic testing of the workforce to inform work assignment decisions?
	for hospitals/ICUs to prohibit a health care worker with increased genetic risk of infection from providing direct patient care?
	for hospitals/ICUs to prioritize health care workers with decreased risk of infection to serve as first responders?
	to use genetic information on health care workers’ susceptibility to COVID-19 to determine the level of personal protective equipment to which they have access?
Public health policies and practices Is it ethically acceptable …	for quarantine measures/policies to be informed by genomics, i.e., those who are at lower risk of contracting the disease do not have to stay at home?
	for school attendance/closure policies to be informed by genomics, i.e., schools can remain open for students and teachers at lower risk?
	for travel and immigration restrictions to be informed by genomics (e.g., super-spreaders face increased restrictions)?
	for vaccine (once available) distribution to be prioritized for those most likely to develop severe disease or least likely to show symptoms (and therefore unknowingly spread disease)?

## Potential uses of genomic information and ethical implications

Identifying genomic variants that contribute to COVID-19 outcomes would offer potentially useful information, especially if the penetrance of these host genetic variants is high. Such information could inform clinical decision making [patient triage, access to or prioritization for particular therapies (e.g., medications, ventilators)], participation in clinical research, workforce requirements, and public health containment practices. However, a number of ethical questions would need to be considered in guiding policy-making in each of these domains.

### Clinical decisions

Essential medical resources such as ventilators, intensive care unit (ICU) beds, and therapies such as extra-corporal membrane oxygenation might become scarce in many areas, particularly in the context of another global wave. Several frameworks have been developed for determining how to allocate under scarcity, focusing on short- and long-term survival, assessments of benefit, illness severity and equity, and prioritizing instrumental value (e.g., those essential to pandemic response such as healthcare workers) [7]. Sometimes, these frameworks conflict with one another, exacerbating the moral distress experienced by frontline workers [8]. In the face of resource scarcity and ambiguous or conflicting guidelines, genetic information could play a role in determining who receives an ICU bed or a ventilator. Similarly, genotypic information might assist in prioritizing access to therapies or vaccines (when they are developed) if they are in short supply. In situations where necessary interventions and facilities are insufficient to treat everyone, genetic information could support the decision to prioritize patients at greatest risk of severe disease to receive care first. Alternatively, scarce resources could be offered to patients whose genetic profile is consistent with a better prognosis for recovery.

### Workforce decisions

In the COVID-19 pandemic, protecting the workforce is critical so they are available to provide necessary services. Genotyping of frontline workers might be used to assign greater responsibility to those whose genotypes indicate a greater resistance to COVID-19 or response to a vaccine. In the specific case of healthcare workers, genotyping might assist in determining who should be first responders, who should be assigned to less exposure-prone environments or duties, or who should remain at home. If personal protective equipment is scarce, healthcare workers may be screened, and practices modified based on genotype. Healthcare workers who knew they were at lower risk of contracting or spreading COVID-19 may be more comfortable entering isolation rooms and providing care to patients. Such decisions based on genotype might have positive effects on patient well-being but may increase the burden on some healthcare workers caring for these sickest patients. Approaches for frontline workers in other high-risk occupations—such as teachers, grocery store workers, and trash collectors—could similarly change based on new understanding of genomic risk.

### Public health practices

Genomic information could be used to identify where vaccines or therapies should be deployed most urgently, and where other public health control strategies to halt the spread of infection—for example case identification and isolation—should be implemented more strictly. Support for infection control and containment measures such as quarantine could be strengthened by the identification of genetic markers that indicate a higher likelihood of transmitting COVID-19 (so-called super-spreaders). Strict containment policies such as “self-quarantine” or “shelter-in-place” are intended to minimize risk of transmission within the population. But such limitations on individual freedom can have negative health consequences. Extreme isolation and loneliness, as well as economic concerns, can contribute to or exacerbate mental health problems and other psychosocial effects. School closures can expose children who rely on schools for physical protection and/or daily nourishment to increased harm. To mitigate such harms, genetic information could be used as a factor in decisions about who to quarantine, helping to selectively exclude teachers and children at increased risk from going to school, but allowing schools to remain open for those at lower risk. Travel and work restrictions might be loosened for individuals whose genotype places them at lower risk of contracting or spreading COVID-19.

## Unintended consequences of using genetic information

While use of genomic information could be ethically justified on grounds of maximizing benefits over harms, potential negative consequences—such as exacerbation of stigma, discrimination, or inequities—must be considered [4, 9]. For example, those who are identified as “super-spreaders” could experience increased breaches of privacy, stigmatization, blame, or legal liability. Furthermore, financing of vaccine or drug development might preferentially support the production of formulations for subgroups with particular at-risk genotypes, creating inequities in access for others.

Additional equity-related concerns would arise if a genetic variant is identified that protects against COVID-19 transmission or disease severity but the prevalence of that variant varies by ancestry. For example, in hepatitis C virus (HCV), the protective IL28b variant is less frequent among people of African descent, and suggestions have been made to ration access to expensive antiviral treatment by IL28b genotype [10]. Genetic information about risk of COVID-19 would exacerbate health disparities if it was used to withhold genomic testing, treatment, or other scarce resources based on ancestry. The stakes are particularly high in the current pandemic, given the disparities with regard to who is contracting and suffering the most severe effects of COVID-19.

## Conclusions and outlook

In short, host genetic studies are *and should be* conducted to improve our basic biological understanding of SARS-CoV-2 infection and to inform the development of public health strategies and policies, targeted therapies, and improved vaccines. However, how COVID-related genomics research is conducted and how the results are used could have unintended consequences that must be anticipated by clinical leadership and policy makers. In parallel with efforts to develop and implement frameworks for distribution of scarce resources, including vaccines, we call for the development of a framework for identifying and addressing ELSI issues when designing and conducting genomic research on host factors and host–pathogen interactions in COVID-19, and in any clinical, workforce, or public health policy decisions that use the resulting data.

## Data Availability

Not applicable.

## Abbreviations

COVID-19: The novel coronavirus disease
ELSI: Ethical, legal, and social implications
ICU: Intensive care unit
HCV: Hepatitis C virus
